# Possible Roles of Cyclic Meditation in Regulation of the Gut-Brain Axis

**DOI:** 10.3389/fpsyg.2021.768031

**Published:** 2021-12-22

**Authors:** Debananda S. Ningthoujam, Nilkamal Singh, Saikat Mukherjee

**Affiliations:** ^1^Department of Biochemistry, Manipur University, Imphal, India; ^2^Department of Yoga, Manipur University, Imphal, India

**Keywords:** yoga, cyclic meditation, brain, autonomic nervous system (ANS), gut-brain axis, microbiome-gut-brain-axis (MGBA)

## Introduction

Yoga is an ancient spiritual practice which originated from India. In recent decades, there has been increasing interest in yoga, mainly because of its applications in health and wellness. Among various techniques of yoga, the practice of meditation has been found to cause several psychophysiological effects. Most meditation techniques are practiced in a stable and comfortable posture but there are also meditation techniques that involve movement. Cyclic meditation (CM)—a technique derived from one of the *Upanishads*—is a moving meditation technique practiced by combining physical postures (*asanas*) with relaxation procedures. The practice of CM starts with a prayer followed by isometric muscle contraction, supine rest, standing at ease, centering by balancing the body weight on the different parts of the feet, bending to the right and then left sides (*ardhakaticakrasana*), forward bending (*padahastasana*), backward bending (*ardhacakrasana*), and supine rest. During the practice, emphasis is given on relaxation and awareness (Nagendra and Nagarathna, 1997). Scientific studies on the effects of practicing CM have reported myriad physiological and mental health benefits (Subramanya and Telles, 2009b).

## Psychophysiology of Cyclic Meditation

A study reported an increase in high frequency component of heart rate variability (HRV) following the practice of CM (Sarang and Telles, 2006b). Another study reported a shift in the sympatho-vagal balance toward parasympathetic dominance during sleep following a day-time practice of CM, while such a change was not observed in the supine rest group (Patra and Telles, 2010). Similar positive findings have also been reported on oxygen consumption and breathing rate by several research groups (Telles et al., 2000; Sarang and Telles, 2006a). One compelling hypothesis about how CM operates is that slow and rhythmic breathing pattern employed during the practice of yoga can activate the vagus nerve. Gerritsen and Band (2018) proposed a two-route model for respiratory vagal nerve stimulation during contemplative practices. One is a top–down route in which low respiration rate and inhalation/exhalation ratio can directly stimulate the vagus nerve and another is efferent vagus nerve activation by upward signals triggered by a state of relaxation and low-threat perception caused by CM. This loop of afferent and efferent vagal nerve stimulation further increases the vagal tone that results in associated beneficial effects.

Besides these findings, CM has also been reported to improve cognitive functions. Cyclic meditation caused an increase in EEG P300 amplitudes in Pz, Cz, and Fz sites and reduction in peak latency (Sarang and Telles, 2006c), besides an increase in Pa wave and Nb wave peak latency and Nb wave peak amplitude of mid-latency auditory evoked potentials (Subramanya and Telles, 2009a). Other studies have associated CM with improved selective attention, concentration, visual scanning abilities, and repetitive motor responses (Sarang and Telles, 2007) and enhanced memory and reduced anxiety (Subramanya and Telles, 2009c).

The above studies were conducted on healthy male participants (age 18–48 years, with more than 3 months of CM practice).

One possible explanation for cognitive enhancements is the activation of the ANS through its projections from the vagus nerve (Benarroch, 1993). Another plausible explanation is that expert yoga practitioners have more widespread functional connectivity within motor, cognitive, and emotional circuits of the brain (Gard et al., 2015). Considering the aforementioned positive effects, we may extrapolate that CM may positively impact the gut-brain axis as stress, anxiety, cognitive functions, and emotional health play critical roles in regulation of the gut-brain axis.

## Gut Brain Axis and Cyclic Meditation

Our brain and gut are intimately connected through the gut-brain axis (GBA). It's a bidirectional link between the CNS and the enteric nervous system (ENS). Enteric nervous system consists of sensory and motor neurons, and interneurons in the wall of the gastrointestinal system. The GBA mediates a complex crosstalk between the gut and the brain sending information to the gut and vice versa. The interaction happens between the endocrine system [hypothalamic-pituitary-adrenal (HPA) axis], the autonomic nervous system (ANS), and the immune system involving cytokines and chemokines. Through this GBA, stress signals from the brain can impact digestion and other physiological functions. Gut microbiota might play major roles in reception, transmission, and modulation of these signals, possibly through the microbiome-gut-brain-axis (MGBA) (Martin et al., 2018).

Gut-brain axis regulates and integrates gut functions and connect emotional and cognitive centers in the brain with the peripheral intestinal functions. Moreover, it regulates enteric reflex, intestinal permeability, immune activation, and entero-endocrine signaling. Communications in the GBA are mediated by several neuro-immuno-endocrine mediators. This bidirectional communication network involves the central nervous system (CNS), the ANS, and the HPA axis. Therefore, both hormonal and neural modes of communication influence intestinal functional effector cells including smooth muscle cells, interstitial cells of Cajal and enterochromaffin cells, immune cells, epithelial cells, and enteric neurons. These same cells are also under the influence of the gut microbiota (Carabottia et al., 2015).

In the last decade, the gut-brain axis has been an important topic of research. Neuroscientists, microbiologists, and nutrition scientists have been exploring how our guts and nervous systems are intimately interconnected, triggering a new paradigm shift in medicine. It is now a scientific consensus that the gut microbiome influences our emotional well-being. This radical thinking has wrought a veritable revolution in medical sciences resulting in the birth of a new discipline called “Psychobiotics” (Skonieczna-Zydecka et al., 2018).

Psychobiotics is an emerging discipline that studies how live microorganisms—bacteria, yeasts, fungi, and viruses—when ingested in adequate amounts confer certain benefits to psychiatric or other nervous disorder patients. These microbes produce neuroactive substances that influence GBA and act like antidepressants. In humans, gut microbiota–brain interactions were first recognized when administration of oral antibiotics rescued patients with hepatic encephalopathy (Foster and McVey, 2013). Microbiota changes have been implicated in anxiety and depression (Naseribafrouei et al., 2014) and autism (Song et al., 2004). Clinical and experimental evidences suggested that the gut microbiota impacts the GBA by directly influencing the CNS functions through metabolic and neuroendocrine pathways.

Gut bacteria helps in development of the CNS and the endocrine system (Stilling et al., 2014) and their absence results in differential expression of neurotransmitters (Stilling et al., 2014), altered gut sensorymotor functions (Iwai et al., 1973), and increased cecal size. Neuromuscular abnormalities, in turn, lead to reduced synthesis and transport of neurotransmitters and muscular contractile proteins (Hooper et al., 2001). Re-establishment of gut microbiota were found to restore the normal physiological functions. Irritable bowel syndrome (IBS) possibly results from an abnormal microbiota causing inflammation resulting in epithelial permeability causing visceral pain and dysregulated ENS (Collins and Bercik, 2009). *Helicobacter pylori*, implicated in gastric ulcer, influences the GBA inducing neurogenic inflammatory processes (Budzyński and Kłopocka, 2014).

Psychological stressors influence the types and biomass of gut microbiota via the ANS (Galley et al., 2014) mediated by secretion of diverse signaling molecules by various effector cells leading to altered gut microbiota. Dysbiotic microbiota can then make humans predispose to inflammation and infections (Hughes and Sperandio, 2008). Moreover, the brain modulates myriad gut functions that includes mucosal immune response; secretion of acids, bicarbonates, and mucus; and gastric motility (Macfarlane and Dillon, 2007).

Stress has been reported to affect the intestinal mucus (Rubio and Huang, 1992), gastric and intestinal postprandial motility (Gué et al., 1989), and ceco-colonic spike-burst activity (Gué et al., 1991). The brain, mediated by altered intestinal permeability, can affect the composition of microbiota. Acute stress results in overproduction of interferon-γ (Demaude et al., 2006) while mild stress cause colonic barrier dysfunction leading to depression as well as vulnerability to colitis (Söderholm et al., 2002). Depression is associated with alterations in colonic motility and intestinal microbial profile (Park et al., 2013).

Stress induced secretion of an antimicrobial peptide, α-defensin, from Paneth cells may cause disruption of gut microbiota (Alonso et al., 2008) which can cause overactivity of harmful bacteria. During surgery, norepinephrine is released that possibly activates *Pseudomonas aeruginosa* leading to gut sepsis (Alverdy et al., 2000), induces proliferation of enteric pathogens and increased virulence of *Campylobacter jejuni* (Cogan et al., 2007), and stimulates the growth of pathogenic *Escherichia coli* strains (Freestone et al., 2003).

These findings strongly suggest that stress response, anxiety, and depression may alter neurotransmitter release which affects the gut microbiota profile. Stress may also disrupt epithelial homeostasis by blocking the protective effects of vagus nerve barrier (Bonaz et al., 2016). Decrease in vagal tone has been implicated in IBD and IBS (Pellissier et al., 2014) and gut microbiome alterations may impact GI motility, integrity, and secretion as well as brain functions by affecting neurotransmission, behavior, and neurogenesis. These influences are, therefore, bidirectional. Cyclic meditation may ameliorate stress induced gut microbiota dysbiosis as CM had been reported to enhance cognitive functions and vagal tone in several studies (Sarang and Telles, 2006a,b,c, 2007; Subramanya and Telles, 2009b). Moreover, Vegan individuals with a long practice of meditation have healthier gut microbiota compared to non-meditators with omnivorous diet. Abundance of bacterial groups such as *Roseburia, Subdoligranulum*, and *norank_f_Lachnospiraceae* are positively related to the number of meditation years (Jia et al., 2020).

Meditation and GBA interrelation is yet to be systematically explored. However, for both humans and animals; certain exercises; and other forms of physical activities have been reported to influence both gut and brain health. Significant inverse relationship exists between physical activity and depression (Parfitt and Eston, 2005) and physical activity and anxiety (Parfitt and Eston, 2005). In rats and mice, it was found that short term exercise protected them against anxiety (Ramos et al., 1997). Animals with intense physical activity showed enhanced acquisition of a task and memory retention (Van der Borght et al., 2006). Exercise may modulate gut microbiota profile which subsequently might influence brain activity (Dalton et al., 2019). Influence of exercise on human gut has been documented in several studies (Mailing et al., 2019). Gut microbiota of professional players had higher relative abundance of certain gut bacterial taxa; athletes showed lower abundance of *Lactobacillus* and *Bacteroides* (Clarke et al., 2014). Women who performed at least 3 h of exercise per week showed increased levels of *Roseburia hominis, Akkermansia muciniphila, Faecalibacterium prausnitzii*; gut bacterial genera known to be producers of butyrate with beneficial effects on the brain (Bressa et al., 2017). Exercise improves mental and neurological health (Stevens et al., 2018) and these beneficial effects could be mediated by gut microbiota. Daily wheel running increased population of butyrate producing bacteria, *Lachnospiraceae*, reducing anxiety-like behavior in mice and butyrate may activate microglial cells, the brain's immune cells (Varela et al., 2015).

Exercise modulates the balance of “good” and “bad” gut bacteria and enhances the diversity of gut microbiota; the breakdown of which may lead to GI and mental stress through the MGBA. Exercise improves the abundance and diversity of gut microbiota genera belonging to *Firmicutes* (a large phylum of beneficial gut bacteria); this, possibly, may be the link between exercise and gut and brain health (Dalton et al., 2019). Several groups have suggested yoga as a therapy for IBS; resulting in improved physical and mental health, possibly through the MGBA (Silva et al., 2020).

We are, therefore, of the opinion that CM could, possibly, stimulate GBA and MGBA resulting in positive effects on gut, brain, immune, and general health.

## Conclusions

As CM can enhance the vagal tone and improve cognitive functions, we may hypothesize that CM can improve the gut-brain axis crosstalk by fine-tuning the modulation of the gut microbiota through the CNS and ANS pathways. There are inadequate data to understand the definitive effects of CM on the gut microbiota. However, it is reasonable to propose that the long-term practice of CM can reinforce healthy gut microbiota as meditation is positively correlated with preponderance of beneficial bacteria such as *Roseburia, Subdoligranulum*, and *norank_f_Lachnospiraceae* (Jia et al., 2020). Hence, we may conclude that practicing CM may help improve the gut-brain crosstalk through various mechanisms including modulation of the gut microbiota. However, as of now, the link between CM and GBA possibly implicating the microbiome is only a reasonable correlation indirectly deduced from the published literature. The definitive connections among cyclic mediation, microbiome, and the gut-brain-axis can only be established by more systematic studies in this emerging area.

## Author Contributions

DSN contributed in preparing the manuscript and generation of hypothesis. NS contributed in hypothesis generation, preparation of manuscript, and coordination among the authors. SM contributed in manuscript preparation. All authors contributed to the article and approved the submitted version.

## Conflict of Interest

The authors declare that the research was conducted in the absence of any commercial or financial relationships that could be construed as a potential conflict of interest.

## Publisher's Note

All claims expressed in this article are solely those of the authors and do not necessarily represent those of their affiliated organizations, or those of the publisher, the editors and the reviewers. Any product that may be evaluated in this article, or claim that may be made by its manufacturer, is not guaranteed or endorsed by the publisher.
